# Sustaining visceral leishmaniasis elimination in Bangladesh – Could a policy brief help?

**DOI:** 10.1371/journal.pntd.0006081

**Published:** 2017-12-12

**Authors:** Alyssa Fitzpatrick, Noor Saad M. S. Al-Kobaisi, Jessica Beitman Maya, Yu Ren Chung, Satyender Duhan, Erdene Elbegdorj, Sushant Jain, Edward Kuhn, Alexandra Nastase, Be-Nazir Ahmed, Piero Olliaro

**Affiliations:** 1 Blavatnik School of Government, University of Oxford, Oxford, United Kingdom; 2 National Institute of Preventive and Social Medicine, Dhaka, Bangladesh; 3 Special Programme for Training and Research in Tropical Diseases, World Health Organisation, Geneva, Switzerland; 4 Centre for Tropical Medicine and Global Health, Nuffield Department of Medicine, University of Oxford, Oxford, United Kingdom; FIND, SWITZERLAND

## Abstract

Bangladesh has made significant progress towards elimination of visceral leishmaniasis, and is on track to achieve its target of less than one case per 10,000 inhabitants in each subdistrict in 2017. As the incidence of disease falls, it is likely that the political capital and financial resources dedicated towards the elimination of visceral leishmaniasis may decrease, raising the prospect of disease resurgence. Policy memos may play a crucial role during the transition of the elimination plan from the ‘attack’ to the ‘consolidation’ and ‘maintenance’ phases, highlighting key stakeholders and areas where ongoing investment is crucial. An example of a policy brief is outlined in this paper. The background to the current elimination efforts is highlighted, with emphasis on remaining uncertainties including the impact of disease reservoirs and sustainable surveillance strategies. A stakeholder map is provided outlining the current and projected future activities of key bodies. Identification of key stakeholders subsequently frames the discussion of three key policy recommendations in the Bangladeshi context for the transition to the consolidation and maintenance phases of the elimination program. Recommendations include determining optimal vector control and surveillance strategies, shifting the emphasis towards horizontal integration of disease programs, and prioritising remaining research questions with a focus on operational and technical capacity. Achieving elimination is as much a political as a scientific question. Integrating the discussion of key stakeholders with policy priorities and the research agenda provides a novel insight into potential pathways forwards in the elimination of visceral leishmaniasis in Bangladesh and in the rest of the Indian subcontinent.

## Author summary

In this article, we examine the challenges of eliminating visceral leishmaniasis in Bangladesh from a policy perspective. Visceral leishmaniasis is a neglected tropical disease which causes significant morbidity and mortality, but recent efforts in Bangladesh, India and Nepal (the countries that used to have two-thirds of all cases in the world) have made significant headway in reducing the extent of the disease. However, ongoing concerted effort is needed to ensure that elimination of the disease is maintained in the long-term. This will require engagement and coordinated effort from a variety of stakeholders, including national and international policy makers, funders and implementers. We present the identified challenges, stakeholder map and potential solutions in the form of a policy brief to distill the key actors and actions that are necessary to maintain momentum towards elimination of visceral leishmaniasis. Such actions include determining optimal long-term vector control and surveillance strategies, and attempting to integrate the visceral leishmaniasis program into existing health systems. The research agenda should also be prioritised, with a focus on addressing operational and technical questions.

## Introduction

The conclusion of 2017 marks the deadline for the achievement of elimination of visceral leishmaniasis (VL; kala-azar) as a public health problem in five South Asian states. Bangladesh, India and Nepal signed a Memorandum of Understanding (MOU) in 2005, seeking to achieve elimination by 2015. This deadline was extended in 2014 to the end of 2017 with the inclusion of Bhutan and Thailand in the MOU [1]. In the context of Bangladesh, elimination as a public health problem is defined as less than one case per 10,000 inhabitants in each upazila (subdistrict) [2]. Of the 489 upazilas in Bangladesh, 100 were reported to be initially endemic for kala-azar [3]. By April 2017 the incidence of VL has dropped below the target in all upazilas, and attainment of the target will hopefully be confirmed at the end of the year [4]. As the elimination efforts shift from achieving the target to consolidating and maintaining progress, it is timely to consider where further action is needed. This paper seeks to present the challenge of consolidating progress towards elimination within the context of a policy framework, analyzing key players and issues where further action is necessary. Box 1 summarises the role and structure of the policy brief.

**Box 1. The role of the policy brief**

Health policy priorities are determined by government departments and funding bodies weighing scientific, political and economic factors. Policy briefs are a valuable tool to assist decision-makers in valuing relevant factors and options, and providing evidence-based recommendations to support the achievement of important public health aims.

Briefs should be concise, action-oriented and synthesize relevant cross-cutting considerations, such as the economic and political impact of decisions. Briefs generally have a set structure as follows:

1. Executive summary and recommendation–a brief, high-level overview of the memo.
2. Background–identification of the key issue, and relevant background information to contextualizes the problem.
3. Stakeholder map–a map outlining the key players and their relative priorities. This map can assist the decision-maker in devising strategy and understanding the implications of decisions.
4. Policy options–a range of policy options, alongside their benefits and disadvantages should be presented.
5. Policy recommendation–a preferred policy and rationale should be offered, outlining the key factors supporting the decision.

Visceral leishmaniasis, or kala-azar, is caused by protozoan parasites from the genus *Leishmania* which are spread by the female sandfly *Phlebotomus argentipes*, and is classified among the neglected tropical diseases [5]. It is characterized by fever, fatigue, weight loss, hepatosplenomegaly, lymphadenopathy and anaemia, with a near universal case fatality without treatment [6,7]. However, the majority of infected subjects are asymptomatic. [6] Among those treated, a proportion will develop post-kala-azar dermal leishmaniasis (PKDL), with the emergence of skin lesions usually six months to three years after the original condition has been cured [6]. In some cases; however, presentation may be delayed or not associated with previous clinical kala-azar.

Modern-day Bangladesh was the site of the first reported outbreak of visceral leishmaniasis, which accounted for 75,000 deaths between 1824 and 1827 [5]. Over the years, a number of attempts have been made to control the burden of visceral leishmaniasis, which disproportionately affects the Indian subcontinent [8]. The Malaria Eradication Program in the 1950s had a positive impact on the control of visceral leishmaniasis in Bangladesh, with only 59 cases reported between 1968 and 1980 [5]. However, subsequent disease resurgence with cessation of widespread spraying of dichlorodiphenyltrichloroethane (DDT) has prompted new and wide-ranging efforts to curb the disease, beginning in 2005 with the signing of the MOU.

A number of factors suggest that long-term elimination of kala-azar may be achievable. These include that in the Indian subcontinent the disease is only hosted by humans, with a single vector and close geographic clustering of cases [9]. The recent availability of a diagnostic test, based on the rk39 antibody, has facilitated improved diagnostic testing in the field. Similarly, the advent of liposomal amphotericin B as a single-dose treatment has provided access to readily available and tolerable treatment [9]. Access to liposomal amphotericin B has been facilitated initially by a 2007 pricing agreement and subsequently in 2011 by a donation agreement between the manufacturer and WHO [9–11]. These factors have supported the development and implementation of the elimination plan currently in place in Bangladesh.

Much as in the other countries of the Indian subcontinent, the national kala-azar elimination program (NKEP) in Bangladesh consists of four discrete phases: a preparatory phase, attack phase, consolidation phase and maintenance phase [12]. The attack phase is defined by widespread indoor-residual spraying, the implementation of integrated vector management, early diagnosis and treatment and close case and vector surveillance [12]. The consolidation phase has been proposed to be distinguished from the attack phase by limited indoor residual spraying around affected cases (the index case approach), and a shift in focus to treatment of co-infection such as HIV and treatment adherence. The maintenance phase has been described as a state of close surveillance with intervention in small regions where outbreaks occur [12]. However, the optimal approach for transitioning from the attack to the consolidation phases remain contested and the subject of inquiry.

After signing the MOU, Bangladesh introduced a national elimination program in 2008 [13]. Key tenets of the program included training health staff practicing in endemic areas, the introduction of the rapid rk39 diagnostic test and free provision of oral miltefosine therapy [13]. Miltefosine is an oral drug taken as a 28-day course to treat visceral leishmaniasis, which has faced deployment issues and is now surpassed by single-dose liposomal amphotericin B [14]. In 2012, the programme also introduced a 12-week oral miltefosine therapy protocol for PKDL patients, replacing the poorly tolerated regimen with sodium stibogluconate [13]. Blanket coverage with indoor residual spraying with deltamethrin and time-limited provision of long-lasting insecticide treated bednets was also implemented in 2011 in the areas of highest endemicity [13]. Implementation research conducted in conjunction with the Special Programme for Research and Training in Tropical Diseases from the World Health Organization (WHO/TDR) informed key strategies in terms of case identification, case management and curtailing transmission. The NKEP has deployed indoor residual spraying with insecticide and case search through house-to-house visits in 60 households around recently reported VL/PKDL cases (the so-called index case search), known as no kala-azar transmission activity (NKTA), although the systematic deployment of this intervention has been conditional to the availability of adequate human and financial resources.

As the attack phase shifts into the consolidation and later into the maintenance phases in Bangladesh, a number of key uncertainties remain which need to be addressed to ensure the continued progress of the elimination program. The current target of <1 per 10,000 cases per upazila was inherited from historical leprosy campaigns and its epidemiological significance for visceral leishmaniasis is uncertain [15]. Modelling studies have sought to delineate whether existing efforts are sufficient to maintain disease control after this target is reached. Seven modelling papers were identified in a recent literature review [8], and more work is underway. However, modelers are struggling with assumptions about the drivers of transmission (symptomatic visceral leishmaniasis patients, asymptomatic carriers, PKDL patients, patients in whom disease reactivation occurs, which is higher among HIV-coinfected patients, and the ‘window of infectivity’ during which a subject is infectious to others), as well as the vectorial capacity of sandflies to transmit the infection [7,16–18]. The relative contribution of these reservoirs remains unclear, and significantly impacts projections regarding the success of control and elimination strategies, especially in the consolidation and maintenance phases.

Detection of asymptomatic cases is currently limited in the field, and treatment is not recommended. The available rk39 test is antibody-based, and therefore unable to distinguish between current and previous infections; in order to diagnose active visceral leishmaniasis it is used as part of an algorithm that requires also presence of fever and enlarged spleen [6]. The availability of cost-effective and field suitable antigenic tests, such as nucleic acid detection tests, would represent a significant advancement [18]. Finally, the optimal methods for disease surveillance and case detection in the consolidation and maintenance phase remain to be determined. The attack and consolidation phases required a targeted, vertical approach (including the ‘fever camp’ and ‘index case’ strategy), but approaches that are more integrated in the general healthcare system are required to make visceral leishmaniasis case detection sustainable and cost-effective in the long-term [19].

Most pressingly, as the incidence and mortality from visceral leishmaniasis falls, donors and local decision-makers may seek to redirect funds elsewhere into more apparently urgent concerns. However, as the experience post cessation of the DDT spraying programme in the 1960s has highlighted, disease resurgence is a real threat which would lay existing efforts to waste. This policy memo therefore seeks to highlight key priorities for decision-makers in managing the transition from the attack to the consolidation phase, including investment in remaining areas of scientific uncertainty which may threaten elimination efforts.

## The policy landscape in visceral leishmaniasis

In this context, it is crucial to highlight the existing stakeholders working towards the elimination of visceral leishmaniasis in Bangladesh. Table 1 classifies the key identified stakeholders as national state actors, intergovernmental organizations and non-state actors, highlights their role during the attack phase and suggests potential activities during the consolidation phase. In Table 2, the stakeholders are mapped according to their primary role in the elimination of the visceral leishmaniasis, demonstrating pertinent interconnections between stakeholders and areas of potential collaboration.

**Table 1:** Detailed stakeholder analysis of key actors in Bangladesh.

STAKEHOLDERS	CURRENT ACTIVITIES	ROLE IN CONSOLIDATION/MAINTENANCE PHASE
**State actors—National**		
Ministry of Health and Family Welfare	▪ In addition to oversight of the activities of the Directorate General of Health Services (see below), the Ministry of Health and Family Welfare acts as the leading body in setting the elimination agenda and coordinating activities of relevant partners	▪ Provide direction and set policy priorities for the consolidation phase ▪ Co-ordinate activities of relevant bodies and foster partnerships with international donor organisations
Directorate General of Health Services	▪ Responsible for epidemiological surveillance of visceral leishmaniasis cases using web-based tools ▪ Provision of vector management with indoor residual spray and long-lasting insecticide treated bednets ▪ Role in co-ordination of active case detection strategies ▪ Conducted clinical trial of combination therapies [20]	▪ Continue to co-ordinate surveillance for outbreaks of visceral leishmaniasis ▪ Co-ordinate treatment for identified cases of kala-azar ▪ Provision of integrated vector management
Ministry of Planning	▪ Approval of operation plan within each 5 year cycle	▪ As per the attack phase
Ministry of Finance	▪ Seek out funding opportunities with international donors ▪ Prioritise pro-poor spending to minimize the burden of kala-azar	▪ As per the attack phase
National Institute of Preventive and Social Medicine (NIPSOM)	▪ Governmental public health teaching and research institution currently engaged in evaluation of visceral leishmaniasis elimination strategies ▪ Current research areas of focus include cost-effectiveness analysis of vector management strategies and monitoring and evaluation tools in Bangladesh [21] ▪ Training of health staff	▪ Continue to conduct research to identify optimal strategies for vector control and surveillance during the consolidation phase ▪ Continue to provide training to health staff, including in integrated approaches to the detection and management of infectious disease
Institute for Epidemiology, Disease Control and Research (IEDCR)	▪ Central public institution conducting large scale research on visceral leishmaniasis ▪ Key areas of research focus have included studies of treatment and detection options for visceral leishmaniasis [21,22] ▪ Training of health staff to meet requirements of national programs	▪ Continue to investigate operationally feasible and cost effective vector control tools, treatment and surveillance approaches in the consolidation phase ▪ Advocate for increased funding to research programs, including partnerships with local universities and medical colleges ▪ Continue to provide training to health staff, including in integrated infectious disease management with other tropical diseases
Bangladesh Medical Research Council, Dhaka	▪ Currently engaged in supporting research into tropical diseases in Bangladesh ▪ Provides training in research methods ▪ Policy advocacy to support investment in key areas of health concern [21]	▪ Advocate for increased funding to support further locally-based research projects into the control of visceral leishmaniasis ▪ Promote ongoing investment and policy development during the consolidation phase of the elimination plan
**Intergovernmental Organizations**		
World Health Organization–Headquarters; South East Asia Regional Office; WHO Country Office—Bangladesh	▪ Promote regional co-ordination between members of the MOU	▪ Provide technical support to national governments in conducting a review of outlined objectives in the national strategies, and facilitate round-table discussion on strategies for the consolidation phase
	▪ Provision of technical expertise and support to local and regional programs. This includes the Regional Technical Advisory Group on Kala-azar Elimination (RTAG) and human resources support for surveillance activities, data management and case management	▪ The current agreement for donation of liposomal amphotericin B expires in 2021 [23]; SEARO must play a key role in continuing to advocate for affordable access to essential medicines beyond this date
	▪ Advocate for and negotiate access to subsidised supply of diagnostic kits and medications e.g. donation of liposomal amphotericin B	▪ Verification of elimination of kala-azar from upazilas
Special Programme for Research and Training in Tropical Disease (WHO/TDR)	▪ Provide technical and financial support to control programme and research institutions for research agenda setting and execution ▪ Development, testing and implementation of adapted tools for case identification, case management and transmission control ▪ Facilitate the research/policy/practice interface ▪ Piloted the introduction of District Health Information System version 2 (DHIS2) in collaboration with Deutsche Gesellschaft für International Zusammenarbeit (GIZ), the German Corporation for International Cooperation	▪ As per attack phase. ▪ Facilitate research into integrated, sustainable approaches
**Non-state actors–including non-governmental organisations and consortiums**		
KalaCORE–consortium formed by Drugs for Neglected Diseases initiative, Mott MacDonald, London School of Hygiene and Tropical Medicine, and *Médecins Sans Frontières [24]*	▪ Provides human resources support to the National Kala-azar Elimination Programme (NKEP) to improve administrative management	▪ The program is due to expire in 2018; should activities continue suggested roles are included below
	▪ Provision of trained health personnel to facilitate programme development including logistics and supply, data management	▪ Facilitate transition to national health system and support strengthening of the health system
	▪ Support training of health staff including management and referral, outbreak management and community education	▪ Facilitate shift towards integration of kala-azar programs with other horizontal healthcare initiatives e.g. leprosy and malaria control strategies
		▪ Ongoing provision of technical support and training
International Centre for Diarrhoeal Disease Research, Bangladesh (Iccdr,b)	▪ Major local research centre conducting research activities on tropical diseases in Bangladesh in collaboration with international partners [21] ▪ Active participant in raising awareness of the disease in endemic communities ▪ Providing technical support for development of web-based surveillance system in collaboration with GIZ (DHIS2) [25]	▪ Continue to strengthen international collaborations to promote locally based research on VL and related tropical disease ▪ Advocate for funding and infrastructure to assist national universities and medical colleges in establishing a robust research program ▪ Continue to engage in community awareness activities to promote engagement with local health services
FIND [26]	▪ Prioritise development of test of cure for VL and PKDL, and appropriate testing strategies in immunocompromised patients	▪ As per attack phase
Japan International Co-operation Agency (JICA) [27]	▪ Co-funded development of Kala-azar Research Centre at the SK Hospital in Mymensingh in collaboration with icddr,b	▪ Continued funding for local research centres to support operational assessments of vector and surveillance control strategies
PATH [28]	▪ Non-profit organisation focusing on global health innovation and providing pharmacovigilance in collaboration with the icddr,b and DGHS for leishmaniasis	▪ Continued role in pharmacovigilance activities

**Table 2:** Stakeholder map by major role.

Role	Key Stakeholders
Agenda Setting	▪ Ministry of Health and Family Welfare ▪ Ministry of Planning ▪ WHO/HQ, SEARO and Country Office—Bangladesh
Funding–Research	▪ Bangladesh Medical Research Council ▪ JICA ▪ WHO/TDR & WHO-HQ
Funding–Execution	▪ Ministry of Finance ▪ WHO–SEARO & WHO-HQ
Research Activities	▪ Icddr, b ▪ IEDCR ▪ NIPSOM ▪ FIND ▪ PATH ▪ WHO/TDR
Execution & Service Delivery	▪ Directorate General of Health Services / Centre for Disease Control ▪ Icddr, b ▪ KalaCORE ▪ WHO-HQ

Abbreviations: HQ-headquarters; Icddr,b–International Centre for Diarrhoeal Disease Research, Bangladesh; IEDCR–Institute for Epidemiology, Disease Control and Research; JICA–Japan International Co-operation Agency; NIPSOM—National Institute of Preventive and Social Medicine; SEARO–South East Asia Regional Office; TDR–Special Programme for Research and Training in Tropical Diseases; WHO–World Health Organisation

## Policy priorities for the consolidation and maintenance phases

As Bangladesh transitions from the attack to the consolidation phase of the visceral leishmaniasis elimination program, the operational plan will also need to adjust to reflect the lower disease burden. The onus for advancing the elimination plan lies with national bodies as some international stakeholders wind down their contribution; where possible, donors must be engaged to continue their involvement to prevent disease resurgence. Importantly, international stakeholders and national bodies should collaborate and coordinate to ensure that adequate capacities remain in the country when international aid ceases. National bodies should emphasise the opportunity cost of disease resurgence as a result of reduced or diverted efforts. Three key policy priorities are identified and explored as central to the transition from the attack to the consolidation phase of the elimination program.

### Determine optimal strategies for transmission control and surveillance in the consolidation phase

As the incidence of disease falls, cost-effective strategies for long-term transmission control and disease surveillance must be established. Blanket provision of indoor residual spraying is unsuited as a long-term control strategy in view of significant cost and resource requirements [29]. Implementation research is contributing new findings suggesting that alternative long-term vector control strategies are feasible. Studies in Bangladesh and elsewhere examine and compare the effects of insecticide treated bednets, wall painting and wall lining, in addition to environmental management. Bednets appear to confer protection, whether insecticide-treated or not [29–31]. A multi-centre cluster randomised controlled trial across Bangladesh, India and Nepal found that insecticide impregnated wall linings had the most sustained impact on sandfly density, surpassing insecticide treated bednets and environmental management; these effects were shown to extend for at least two years in a cluster-randomised study in Bangladesh in terms of sandfly reduction and mortality [31, 32]. Within three months after the intervention, another cluster randomized study found that wall painting was more effective in reducing sandfly density than insecticide spraying, wall lining and bednets [33]. Combination approaches are likely to prove effective in the long-term, with a recent study identifying that long-lasting insecticide treated bednets in combination with outdoor spraying of breeding areas yielded the highest reduction in sandfly density compared with alternative combined modalities [34]. With this information and other studies underway, Bangladesh must now transition from the indoor residual spraying-led approach of the attack phase to one which more sustainably utilises these novel control strategies for the consolidation phase, while evaluating long-term effects and costs.

Bangladesh to date has also effectively utilised active case detection strategies in the attack phase, searching houses for suspected cases during indoor residual spraying in preselected hyperendemic villages (‘blanket search approach’), or in the fifty houses surrounding any case who presents to the health centre (‘index case approach’) [20]. The former yields higher numbers of new cases (during the attack phase) but at greater effort and cost than the index search approach [19, 35]. The latter is likely to represent the most appropriate search strategy as disease incidence falls, provided cases are identified soon enough to limit the number of secondary infections generated [19]. However, a disturbing finding is that the median time from onset of symptoms to treatment during the attack phase was 78 days in Bangladesh, essentially due to patient’s healer shopping practices and healers failing to diagnose and refer suspected cases, and had not improved from the 58 days estimated in a previous cross-sectional study in 2007 [36, 37]. With declining incidence and awareness these delays are even likely to increase.

### Horizontal integration and intervention packages

The ‘fever camp approach’ has also been evaluated as an active detection strategy in the Indian subcontinent. In initial studies, the fever camp demonstrated higher sensitivity in detecting clinical cases than alternative approaches at a greater overall cost but lower cost per case in the Bangladeshi context [19, 35]. However, as the incidence of cases falls, the experience in India has demonstrated that the cost per case will rise markedly [35]. In order to improve cost-effectiveness, a combined fever camp approach assessing for a number of infectious diseases has been proposed. One study evaluated combined camps assessing for visceral leishmaniasis, tuberculosis, malaria and leprosy in Bangladesh, India and Nepal. While this small pilot study could only detect a small number of new cases, the approach was acceptable to the community, with a cost of USD232 per case detected, and provided an opportunity for additional interventions, such as bednet impregnation and community education [38].

Case detection and transmission control activities can also be combined. Various types of index case-based intervention packages are being tested in Bangladesh. These include fever camps at the village of the index case looking for cases of visceral leishmaniasis and PKDL as well as other febrile illnesses, combined with installation of insecticide-impregnated wall lining or impregnation of existing bednets [39].

The assessment of multiple diseases in the combined camp approach highlights the need for Bangladesh to horizontally integrate its visceral leishmaniasis elimination program with other health activities. This may be achieved at the level of the health service, but also through integration with other private entities operating in Bangladesh. By way of example, the Bangladesh Rural Advancement Committee currently offers pathology services for malaria and HIV testing, promotes the use of bednets and utilises community health volunteers to administer health interventions, and could be readily collaborated with to expand access to services for visceral leishmaniasis [21]. The web-based surveillance system DHIS2 by GIZ has the potential to be expanded to other infectious diseases and presents a powerful tool in the management of disease outbreaks. It has significantly improved disease surveillance, making routine data available through public servers and providing real-time information from public health facilities to the Ministry of Health and Family Welfare [40]. The system leverages the government’s existing web-based platform for maternal and child health services to maintain clinic information on patients with visceral leishmaniasis and improve follow-up [25]. Horizontal integration with the treatment of other infectious diseases will improve service delivery and may also engage donors and international stakeholders in refining models of care. This will necessitate health system strengthening including ongoing educational activities by icddr,b and the Directorate of General Health Services. The provision of technical advice by partners regarding health system strengthening may also be of benefit.

### Prioritise remaining research questions

As highlighted earlier in the paper, there are a number of outstanding questions that require continued investments in research to ensure lasting visceral leishmaniasis elimination. This is indeed a hard sell, at a time when the feeling is that the goal is reached, and decision-makers start planning for divesting. However, without filling critical knowledge gaps and innovative tools and approaches, the remarkable achievements of the attack phase are vulnerable to the disease returning in the medium to long-run.

Research has provided the interventions that are being used now and made it possible to eliminate visceral leishmaniasis. This includes the development of tools for diagnosing (the rK39 rapid diagnostic test) and treating the infection (miltefosine, liposomal amphotericin B, paromomycin), as well as implementation research that identified effective ways of conducting active case identification and reducing transmission through vector control and community participation. The contribution of many actors, both in the countries and internationally, should be acknowledged.

However, as pointed out above, some of these tools and approaches may not work or may not be sustainable in the consolidation and maintenance phases. In particular, the WHO Special Programme for Research and Training in Tropical Disease (WHO/TDR) has supported for more than a decade the joint work of disease control managers and researchers from Bangladesh, India and Nepal to identify research questions and conduct the research to inform policy decisions in terms of transmission reduction, vector control and case identification and management. This group highlighted knowledge gaps and research investment needs ranging from research and development of new tools to intervention and implementation research [41,42]. While a deeper understanding of the epidemiology and transmission of visceral leishmaniasis is needed, improvement in diagnosis, treatment and prevention is also pressing in the transition between attack and consolidation phase. Importantly, this group emphasizes the need to reconsider a target based on reducing the incidence (rather than the prevalence) of visceral leishmaniasis, and maintaining zero transmission in the areas that have achieved the elimination target [42]. These analyses should inform decisions by international and local actors, and be coordinated across the concerned countries of the Indian subcontinent.

Achieving elimination of visceral leishmaniasis is as much a political as a scientific question. As Bangladesh moves towards the consolidation phase of its elimination program, national stakeholders must continue to engage donors and international bodies in coordination with the other concerned countries of the Indian subcontinent to ensure that the long-term goal of elimination is achieved, and resources are not prematurely redirected. The key focus will be on evolving the operational plan towards more sustainable surveillance and vector control strategies, horizontal integration of health resources, and addressing remaining development and implementation research questions to assist in achieving long-term elimination of visceral leishmaniasis.
